# Study on the Formation Mechanism of Cutting Dead Metal Zone for Turning AISI4340 with Different Chamfering Tools

**DOI:** 10.3390/mi13071156

**Published:** 2022-07-21

**Authors:** Shujing Wu, Dazhong Wang, Jiajia Zhang, Alexey B. Nadykto

**Affiliations:** 1 School of Chemistry and Chemical Engineering, Shanghai University of Engineering Science, Shanghai 201620, China; wdzh168@126.com (D.W.); jiajiaz@21cn.com (J.Z.); 2 Department of Cutting Tools and Machining Technologies, Moscow State University of Technology Stankin, Moscow 127055, Russia; nadykto@gmail.com

**Keywords:** chamfered tool, dead metal zone (DMZ), finite element method (FEM), AISI4340, wear

## Abstract

Tools with chamfered edges are often used in high speed machining of hard materials because they provide compelling cutting toughness and reduced tool wear. Chamfered tools are also responsible for the dead metal zone (DMZ). Through numerical simulation of orthogonal cutting with AISI 4340 steel, this paper examines the mechanism of the DMZ, the cutting speed, the impacts of the chamfer angle, and the coefficient of friction on the generation of the DMZ. The analysis is based upon the Arbitrary Lagrangian-Eulerian (ALE) finite element method (FEM) for the continuous process of chip formation. The different chamfered angles, cutting speeds, and friction coefficient conditions are utilized in the simulation. The research demonstrates that a zone of trapped material called DMZ has been formed beneath the chamfer and serves as an effective cutting edge of the tool. Additionally, the dead metal zone DMZ becomes smaller while the cutting speed increases or the friction coefficient decreases. The machining forces rise with increasing chamfer angles, rise with increasing friction coefficients, and fall with increasing cutting speed in both the cutting and thrust directions. In this paper, the effect of different chamfering tools on AISI 4340 steel using carbide tools in the simulation environment is studied. It has certain reference significance for studying the formation mechanism of the dead zone of difficult-to-machine materials such as AISI4340 and improving the processing efficiency and workpiece surface quality.

## 1. Introduction

Due to its high impact resistance and wear resistance, AISI 4340 has been widely used in aircraft take-off, landing gears, and high-powered transmission gears. During high-speed machining, friction is more important to increase the temperature between the tool and the workpiece. Workpieces and cutting tools increase wear rates and shorten tool life due to higher operating temperatures [1]. In response, Jiang [2] used finite element method (FEM) simulations to study the performance of different wiper geometries in the machining of AISI 4340 steel. Simulate the cutting process using the Arbitrary Lagrangian-Eulerian (ALE) method in Abaqus (6.0, Dr. David Hibbitt, Dr. Bengt Karlsson, and Dr. Paul Sorensen, Johnston, Rhode Island, United States). The purpose was to investigate the effect of wiper tool geometry on the cutting performance of AISI 4340 steel, compared to conventional steel tools. The simulation results show that the wiper tool can increase the cutting force and peak cutting temperature, but it can reduce the distribution temperature of the cutting edge, which is beneficial to reduce the wear of the tool. At the same time, the wiper edge also has a great influence on the shape and shape of the chip [3].

The optimization of cutting tool geometry is referred to as edge preparation. Four major types of edge preparation exist in metal cutting: sharp edge, hone edge, chamfered edge, and double chamfered edge. The sharp edge is keenness and cuts easily but can collapse soon. The main function of the chamfered edge is to enhance the cutting edge and reduce the tool wear. Much research [4,5] has been conducted about how the edge geometry of tools influences the cutting process, analytically and experimentally. Research in cutting mechanics has mainly concentrated on machining with tools with sharp edges. Merchant [6] proposed a classical shear plane model and suggested that the chip is created by a shearing process along a shear plane ahead of the cutting edge, moving toward a free surface of the workpiece. Oxley [7] presented a shear zone model by taking into account the impacts of strain, strain rate, and temperature regarding flow stress of the workpiece. El-Wardany [8] suggested that when cutting speed rose, the penetrating depth of the serrated layer and the magnitude of residual stress on the machined surface would rise for the honing tools.

An analytical model is presented to predict residual stresses in the orthogonal machining of AISI4340 steel [9]. The novelty of the model lies in the physics-based approach, focusing on the nature of the contact stress in different processing regions and the effect of the processing temperature. Turning of AISI 4340 material was performed using uncoated tungsten carbide with different cutting parameters [10]. This study investigates the cutting performance and comparative evaluation of the machinability improvement of nanofluids for minimal quantity lubrication (MQL) technology during hard turning using four different compositions of high-strength low-alloy AISI 4340 steel. Cutting force, tool wear, surface integrity, and chip morphology were considered as process performance characteristics for evaluating the machinability of quenched AISI 4340 steel [11].

Many researchers [12,13,14,15,16] have observed this stable trapped work material zone, which they have named the DMZ. The built-up-edge (BUE), which emerged before the tool, is distinct because it is not a stable structure and will eventually break up [17]. To model the continuous process of chip production when cutting with chamfered tools, Movahhedy [18] proposed a numerical analysis based on finite elements. They discovered that the chip removal procedure is not considerably affected by a stagnant material zone beneath the chamfer angle. Ozturk [19] employed a quick stop device to collect chip root samples that are typical of the deformation occurring under dynamic cutting scenarios. Additionally, they did a metallographic analysis on selected specimens to examine the microstructure and creation of the dead metal zone. By examining the variation of the dead metal zone with the computer aided quick-stop device (CAQSD), they discovered that the dead metal zone grows as the cutting-edge radius grows [20] from the experimental photomicrographs. Karami [21] used physical modeling tests and finite element analysis to examine the extrusion of rectangular, square, and L-shaped sections. They explored how the shape complexity influenced the material flow and the dead metal zone formation under various situations. From the experiment, Uysal [22] observed that the dead metal zone shrank as the flank-wear rate rose but its size grew as the cutting edge radius increased. The examination also covered cutting force and thrust force. It was established that both forces increased as the cutting-edge radius and flank-wear rate increased.

Based on the above, this paper will focus on the formation mechanism of DMZ in the process of cutting AISI 4340, as well as the influence of chamfering angle, cutting speed, and friction coefficient on the formation of DMZ, etc., to lay a foundation for improving the application of chamfering tools in cutting difficult-to-machine materials. This study will examine the effect of cutting speed, DMZ friction coefficient, and chamfer angle on cutting force, and numerically analyze DMZ creation using the ALE method and various chamfering tools based on the finite element method. At the same time, the effect of different chamfering tools on AISI 4340 steel using carbide tools in the simulation environment is studied. It has certain reference significance for studying the formation mechanism of the dead zone of difficult-to-machine materials such as AISI4340 and improving the processing efficiency and workpiece surface quality.

## 2. Numerical Simulation

### 2.1. Numerical Approach

In-depth examination of the process variables, such as strain, stress, velocity, and temperature, which are very challenging to detect experimentally, may be achieved using numerical modeling of the cutting process and the use of complex material and friction models. However, the conditions that exist in the small shear zone and at the chip-tool interface, where materials are severely deformed at high strain rate and temperature, are exceedingly complex, making it difficult to simulate the cutting process.

There are two main traditional element approaches named the Lagrangian formulation and the Eulerian formulation in numerical simulation of cutting. The Lagrangian formulation can simulate the whole process of machining and better predict the shape of chip and the distribution of stress, strain, and temperature, but the separation line and the chip separation criteria should be defined beforehand. While the chip separation requirements are not required, the Eulerian formulation is only applied when the cutting has reached a steady state and both the shear angle and chip shape are known. The mesh should be fixed to avoid the misconvergence caused by serious distortion since metal cutting is a large deformation, high strain rate, and thermal coupling process. An alternate strategy that eliminates mesh distortion in the Eulerian formulation, combines the advantage of both approaches, and models the unconstrained flow of chips in the Lagrangian strategy should be available. Therefore, Gadala [23] reported an approach named ALE formulation. The analyst specifies an independent and arbitrary motion for the ALE formulation. The impact of the chamfered tool on the process of chip production and the generation of dead metal zones is examined in this article using the ALE method.

### 2.2. Orthogonal Cutting Model with Chamfered Tool

**Figure 1:**
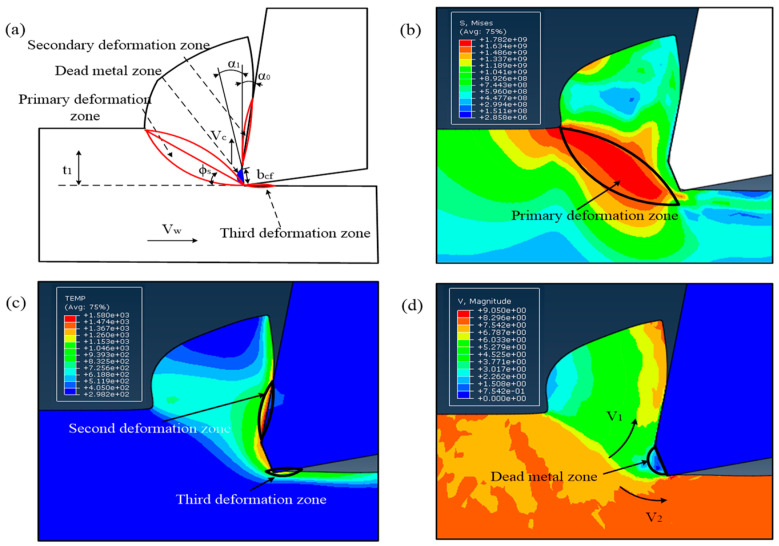
Schematic view of metal cutting process with a chamfered tool. (**a**) Dead metal zone (DMZ); (**b**) Stress contours; (**c**) Temperature; (**d**) Velocity.

A force modeling approach to include the dead metal zone effect based on a three-zone model and investigated the forces in orthogonal cutting with chamfer tools. Long [24] presented a novel force modeling method to analyze the forces in orthogonal cutting with chamfer tools and incorporate the dead metal zone effect based on a three-zone model. He discovered that the dead metal zone largely contributes to the thrust force, whereas the primary and secondary deformation zones primarily influence the cutting force. With the help of this discovery, we can comprehend the DMZ’s creation mechanism better. The orthogonal metal cutting procedure with a chamfer tool is schematically shown in Figure 1a. This model first proposed by Ren [25] and then improved by Wan [16] is composed of four deformation zones: the primary, secondary, third deformation zones, and the dead metal zone.

The simulation contours as shown in Figure 1b stress contours, Figure 1c temperature, and Figure 1d velocity are also confirmed the theory. Due to the material’s strong extrusion and significant deformation, as illustrated in Figure 1b, the location of stress concentration is the primary deformation zone. Due to friction in these areas, Figure 1c demonstrates that the second and third deformation zones have the highest temperatures. In order to prevent the tool’s surface from wearing under various types of heavy cutting conditions, Figure 1d shows that a portion of the workpiece material becomes trapped and stagnant under the chamfer. At the front of the chamfer, material flow simultaneously divides into two sections, one of which turns to the chip at a speed of V1, and the other of which creates the machined surface at a speed of V_2_.

**Figure 2:**
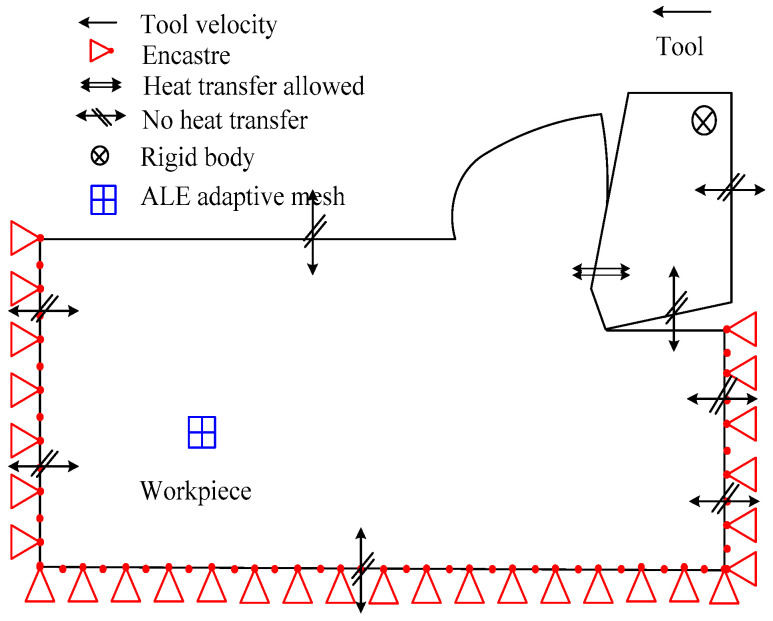
Description of the ALE model.

As shown in Figure 2, this paper uses an orthogonal cutting model, which is based on ALE approach. The rake angle of the tool is 10° and its clearance angle is 10°. The FEM comprises a stiff tool meshed with 3-node elements and a deformable workpiece meshed with 4-node temperature-displacement coupled quad-dominated elements. The approach uses a relatively fine grid division in the cutting area of the workpiece and the area close to the tooltip while using a relatively sparse grid division in the area far from the cutting area and the tooltip. This considerably increases grid division speed while simultaneously ensuring accuracy.

The workpiece is made of AISI 4340, a material whose coefficient of linear thermal expansion varies with temperature. The tool is manufactured by Carbide and uses rigid body restrictions; its deformation is not taken into account and only the influence of temperature is taken into account. The cutting time, which is 0.0008 s, is based on the explicit thermal stress coupling algorithm, which uses surface-to-surface contact, finite sliding, and the kinematic contact method. The cutting tool serves as the main surface, and the surface of the workpiece’s cutting area serves as the second surface. On the bottom edge, left side, and left side of the workpiece, the horizontal displacement boundary conditions are applied. Additionally, the tool is subjected to the cutting speed through a curve of the Tabular Amplitude type.

### 2.3. Model of Johnson–Cook Material

The material constitutive relation that formed by temperature, strain, and strain rate responds to the thermal dynamic state of the material flow stress in certain micro-structure.

It is crucial to consider how the large strain of the material, strain rate, and large amplitude temperature variation affect the flow stress when reflected in the high speed deformation of the material. Strain hardening, strain rate hardening, and softening temperature all affect the Johnson–Cook flow stress model. In this paper, the modified Johnson–Cook model is used as follows [12].

(1) $$\sigma =\left(A+B\varepsilon^{n}\right)\left(1+C\ln\left(\frac{\varepsilon^{1}}{\varepsilon_{0}^{1}}\right)\right)\left(1-{\left(\frac{T-T_{room}}{T_{mwlt}-T_{room}}\right)}^{m}\right)$$

where σ represents the equivalent stress, A represents the initial yield stress, B represents the hardening modulus, C represents the strain rate dependency coefficient, ε represents the plastic strain, $\varepsilon^{1}$ represents the strain rate, $\varepsilon_{0}{}^{1}$ represents the reference strain rate, T represents the operating temperature, $T_{room}$ represents room temperature, $T_{melt}$ represents the melting temperature, n represents the strain hardening exponent, and m represents the thermal softening coefficient. The effective temperature range of the Johnson–Cook model is between room temperature and melting temperature. The workpiece and tool material’s mechanical and thermal properties are taken from reference [17] and are shown in Table 1.

Due to the energy turning into heat during plastic deformation of the high strain rate material, this is frequently accompanied by a rise in temperature. The plastic deformation dissipates as heat in around 90% to 100% of the materials.

## 3. Experimental Work

**Figure 3:**
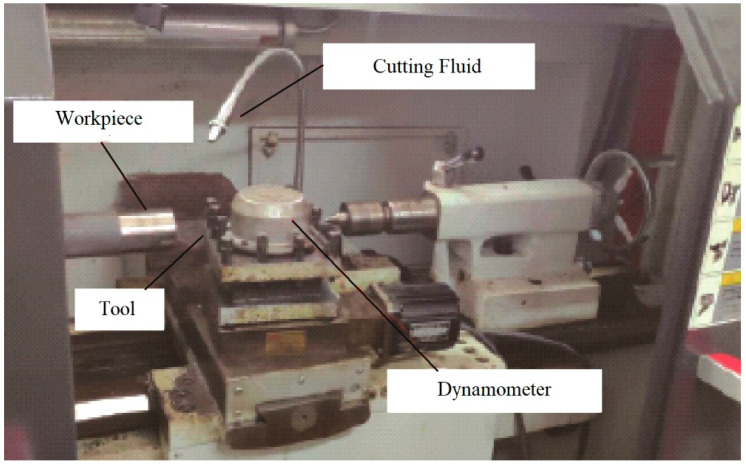
Experimental apparatus for measuring the cutting and thrust force [26].

Ren [25] performed a series of orthogonal cutting tests using chamfered carbide and CBN tools in the past. Cutting forces were measured in each scenario and on a CNC turning center, AISI 4340 disks were turned into plunge orthogonal mode. The first series of tests has various chamfer angles α_o_ and α_1_ and lengths b_cf_ as shown in Table 2. The back engagement is 0.2 mm, the knife step program feed is 0.18mm, and the diameter of the workpiece is 62 mm. In the initial round of testing, the tool’s main rake angle was 10°. In the second round of testing, CBN inserts with AISI 4340 disks were cut at differing rotating speeds using main rake angles of 10° and chamfer angles of −25° and −10°. In every instance, the produced chip was continuous. Figure 3 depicts the experimental apparatus for measuring the cutting and thrust force.

## 4. Results and Discussion

### 4.1. Influence of Chamfered Angle on Chip Formation Process

**Figure 4:**
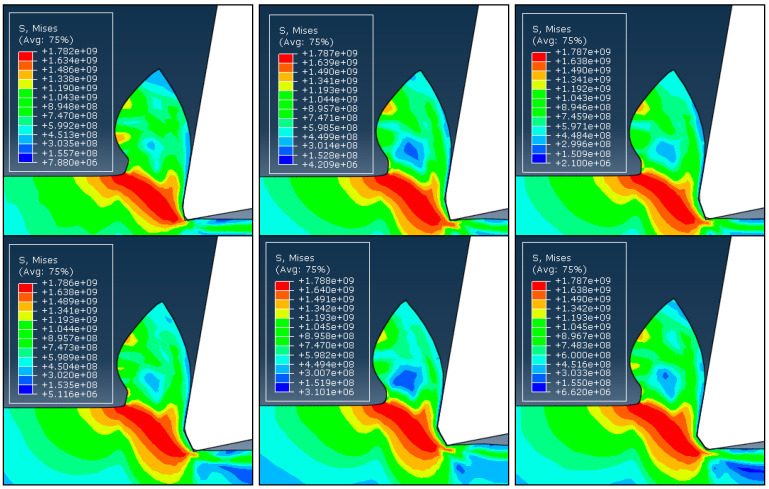
The distribution of stress with different chamfer angles. From top and left: −10°, −15°, −20°, −25°, −30°, and −35°.

**Figure 5:**
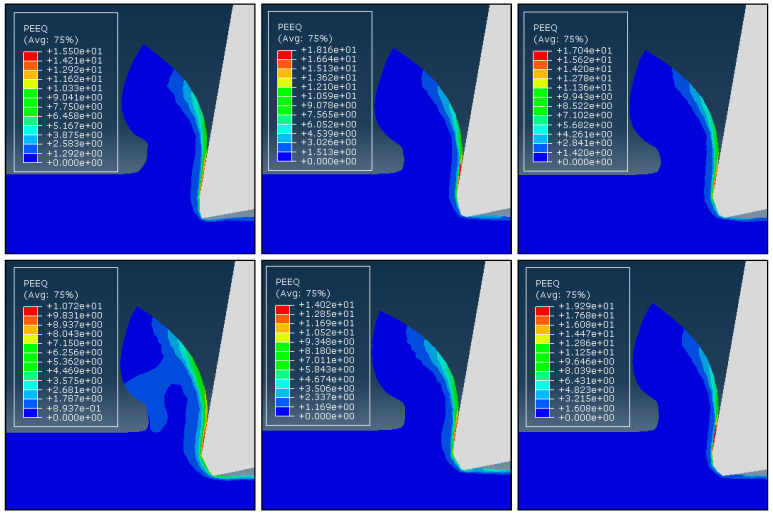
The distribution of strain with different chamfer angles. From top and left: −10°, −15°, −20°, −25°, −30°, and −35°.

By comparing the outcomes of cutting simulations where cutting circumstances are kept constant, but chamfer angles are altered, the impacts of the chamfer angle on the chip generation process are examined. Six chamfer angles of −10°, −15°, −20°, −25°, −30°, and −35° are modeled and compared with each other. Figure 4 and Figure 5 show the distributions of the effective stress and effective plastic strain rate contours for six different chamfer angles under the same cutting circumstances, illustrating how chamfer angle affects cutting variables. Figure 4 makes it clear that the shear zone has a triangle form, spreading from the tooltip and widening toward the free surface at the root of the chip. As the material advances into the center of the shear zone owing to strain and strain rate hardening, the effective stress showed the combined effects of strain, strain rates, and temperature and increased until it reached a maximum at the center of the shear zone. Where the material is softened by the large temperature rise, the stress has lower values near the tool edge than it does in the center of the shear zone. All of the examples exhibit essentially the same chip formation and stress distributions. In the meantime, it appears that the creation of the DMZ has no impact on the growth of the chips or how the stress is distributed.

According to Figure 5, the strain rate distribution has substantially smaller values in other areas than the tool’s flank and the lower portion of the secondary rake face, where very large values can be seen. Effective stress and strain rate distributions exhibit low chamfer angle dependence. In all cases, the primary shear zone’s size and shape, as well as the stress values, are essentially identical. Some experimental findings made by Hirao [27], who observed that the chips generated with varied chamfer angles are essentially the same, support this independence of the cutting process from the chamfer angle. This is due to presence of the DMZ, which fills the chamfer and makes cutting for various chamfer angles almost equivalent.

### 4.2. Effects of the Tool Geometry on Machining Forces

**Figure 6:**
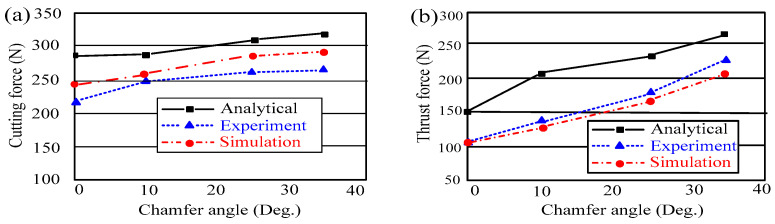
Comparisons of (**a**) cutting force and (**b**) thrust force between analytical, experimental, and simulation results with different chamfer angles [25].

Although the stress distribution and chip formation appear to be unaffected by the chamfer angle, the cutting force is significantly affected. The cutting and thrust forces from experiments and finite element simulations are compared in Figure 6 [18]. The analytical force predictions of Ren [25], who employed a slip-line field solution for the chamfered tools, are also depicted in the figure. Although there are significant differences between the analytical, simulated, and experimental results, they all provide a clear description of the forces’ tendencies that is qualitatively consistent with the others. Additionally, the cutting forces produced from simulation are, on average, about 10% greater than experimental values. The bigger the chamfer angle, the more material from the workpiece that has accumulated in the dead metal zone is strained, which results in an increase in the cutting and thrust forces. However, a number of experimental studies [28,29] reveal that the chamfer angle has a significantly greater impact on the thrust force than the cutting force.

### 4.3. The Existence of the Dead Metal Zone

**Figure 7:**
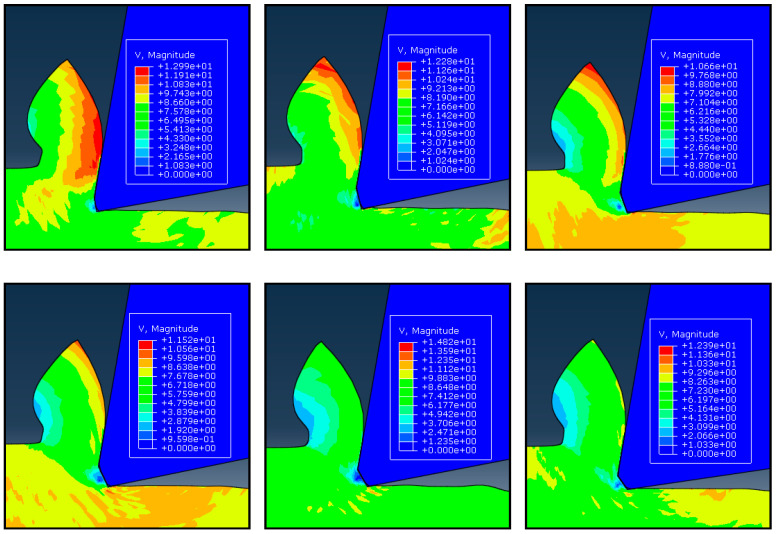
Velocity fields of the simulation with different chamfer angles. From top and left: −10°, −15°, −20°, −25°, −30°, and −35°.

Earlier simulation results [16] show that the chamfered tool holds the largest DMZ than the sharp tool, hone tool, and double tool. Because the negative rake angle can help trap the workpiece material in front of the chamfered edge. According to Movaheddy [18], the DMZ is present while using chamfered tools for cutting. This paper focuses on the DMZ under the chamfered tool and how it is affected by rotating speed and friction coefficient. According to the simulation results, the DMZ always lies beneath the chamfer of the tool, regardless of the cutting circumstances. It nearly entirely fills the tool’s missing nose and serves as its actual cutting edge. By simulating the velocity field and process variables of the work material at the tool edge, it is possible to investigate the possibility of such a DMZ. The velocity field for various chamfer angles is shown in Figure 7. It can be seen that under every chamfer, a portion of the workpiece material is trapped and stays stagnant. The material in the trapped region has a significantly lower velocity than the cutting speed. In this paper, the trapped region’s velocity is only approximately 10% of the cutting speed. The trapped workpiece material region is tiny and depends on the chamfer angle, as indicated by the dark blue color in the contour plots. With the exception of its lower portion on the flank radius, this zone almost completely covers the missing nose, and its shape is compatible with quick stop testing of the Hirao [28]. Cutting with DMZ under chamfer may have many drawbacks, such as increasing the forces and influencing the validity of simulation results. Finding the optimum chamfering structure and studying the DMZ with chamfer tools in orthogonal cutting are important. As shown in Figure 7, the size of DMZ with −25° and −30° chamfered tools are much bigger than others. While the cutting force is increased with the increase of the chamfer angle, the contact length between tool and workpiece of the little chamfer angle tool is short and can lead to bad heat dissipation. It is stated between 15° and 20° is the ideal chamfer angle. The DMZ shrinks significantly as the calculation progresses, demonstrating its form and size instability, which is in good agreement with Jacobson and Wallén [30].

**Figure 8:**
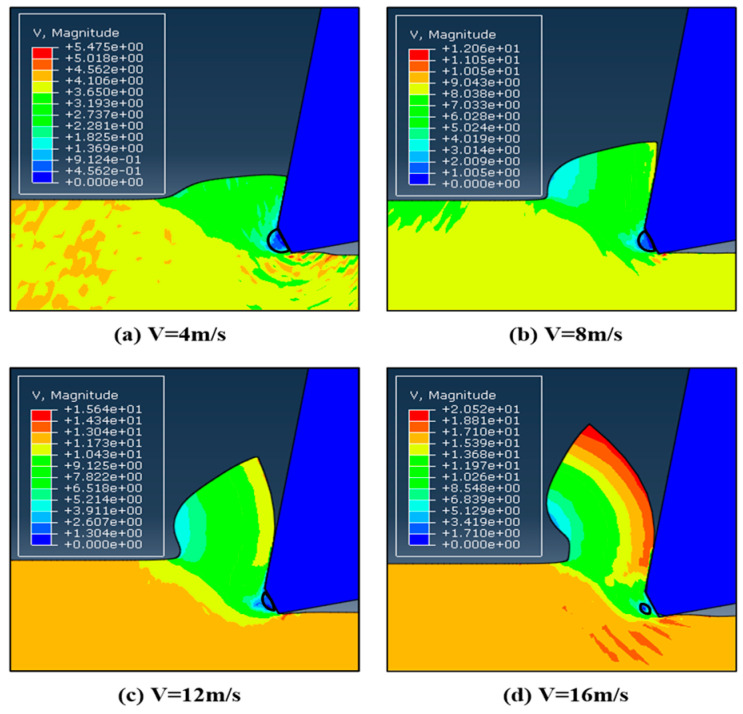
Velocity fields of the simulations with the −25° chamfered tool.

When employing an ALE method with a −25° chamfer tool, Figure 8 depicts the velocity field of the material at various cutting speeds. It can be observed that behind every chamfer, a portion of the workpiece material is trapped and stays stagnant. Specifically, the material in the trapped region moves at a pace that is substantially slower than the cutting speed. The trapped workpiece material region is small and slightly dependent on the cutting speed as seen by the contour plots’ dark blue color. The simulation findings show that the DMZ shapes at the four different cutting speeds are nearly identical. DMZ in Figure 8a,b is significantly larger than those in Figure 8c,d, indicating that the size of the DMZ decreases as the cutting speed increases. When the cutting speed is sufficiently high, the DMZ almost disappears because the heat from the high speed can soften the material close to the edge of the tool.

### 4.4. The Effect of Rotating Speed on Machining Forces

**Figure 9:**
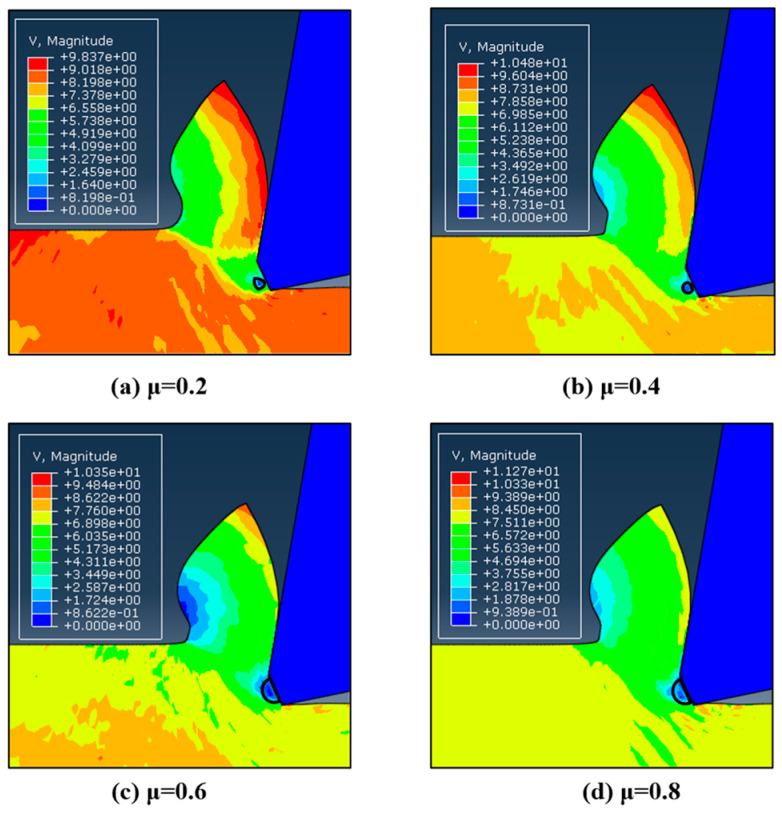
The distributions of velocity fields under different friction coefficient conditions.

**Figure 10:**
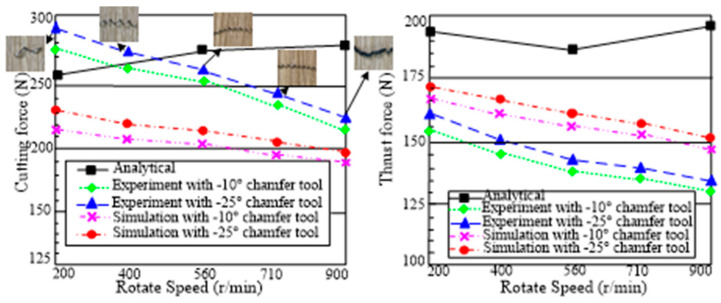
Effect of rotating speed on the cutting and thrust force. Numerical predictions are compared with experimental results and analytical results of Ren [25].

This paper uses −25° and −10° chamfering tools for orthogonal cutting to study the effect of rotational speed on the machining force of the chamfering tool. Five different rotating speeds of 200, 400, 560, 710, and 900 r/min are tested and the pictures of the chip under different speeds are shown in Figure 9, respectively. It is shown the crimp ratio of the chip improves as the rotating speed increases. Additionally, when the rotating speed is sufficiently high, the color of the chip changes because the heat from the high speed can soften the material close to the edge of the tool. In this investigation, using the FEM, the same speeds are simulated with a −10° and a −25° chamfer. The simulation findings are compared with the experimental and analytical results produced by Ren [25], which are depicted in Figure 10. It can be seen that the simulations, which are in good agreement with the experiment results [31], show a declining trend for both the cutting and thrust force. It might be explained by the fact that the DMZ is unstable during high-speed cutting, and that it usually disappears as a result of the heat produced during the cutting process, which softens the material. Due to the presence of the DMZ, the speed has a greater impact on the thrust force than the cutting force even though both forces are impacted by the rotating speed, which is in good agreement with the result reached by Long and Huang [24]. An average of about 24% separates experimental and simulated cutting forces. The analytically anticipated thrust forces, however, deviate from the experimental values with greater disparity.

### 4.5. The Effect of Friction Coefficient on Machining Force

**Figure 11:**
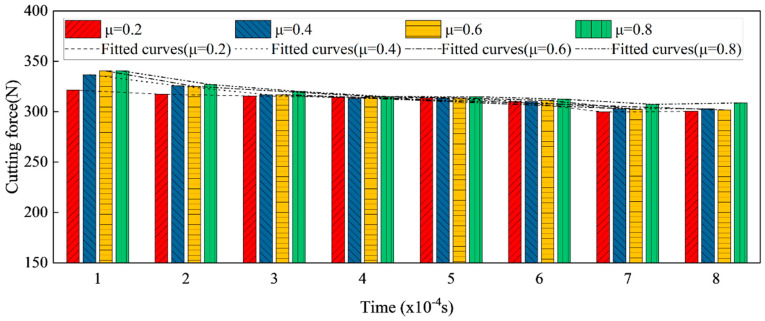
Comparisons of cutting forces with −25° chamfer tool under different friction coefficient.

As the friction coefficient ranges from 0.2 to 0.8, Figure 11 shows the simulation result of cutting force with a −25° chamfer. It is evident that the cutting force is continually changing, but that it always rises as the friction coefficient rises at each time. The aforementioned simulation results support Atlati’s conclusion [32] that the cutting force increase while the friction coefficient increases.

## 5. Conclusions

In this study, the effect of different chamfering tools on AISI 4340 steel using carbide tools was investigated in a simulated environment. The formation mechanism of dead zones in difficult-to-machine materials such as AISI 4340 is discussed. The results are as follows.

(1) Effects of the tool geometry on machining forces. Although the stress distribution and chip formation appear to be unaffected by the chamfer angle, the cutting force is significantly affected. The bigger the chamfer angle, the more material from the workpiece that has accumulated in the dead metal zone is strained, which results in an increase in the cutting and thrust forces.

(2) When simulating with different chamfer tools, depicts the velocity field of the material at various cutting speeds, a portion of the workpiece material is trapped and stays stagnant, the material in the trapped region moves at a pace that is substantially slower than the cutting speed. The simulation findings show that the size of the DMZ decreases as the cutting speed increases. When the cutting speed is sufficiently high, the DMZ almost disappears because the heat from the high speed can soften the material close to edge of the tool.

(3) A study of the effect of rotational speed on the cutting force of a chamfering tool showed that as the rotational speed increased, the chip crimp rate increased, and the color of the chip changed because the heat generated at high speeds softened the material close to the edge of the tool. DMZ is not stable during high-speed cutting and usually disappears due to the heat generated during cutting softening the material. In addition, the research results also show that the cutting force will also increase with the increase of the friction coefficient. Therefore, the friction coefficient should be controlled during the cutting process to reduce the cutting force and cutting temperature and reduce the generation of DMZ.

## Figures and Tables

**Table 1:** Material properties and cutting conditions for the process simulations [17].

Abaqus/Explicit			
Material properties	Plasticity, Johnson-Cook law	A(MPa)	1150
		B(MPa)	739
		n	0.26
		C	0.014
		m	1.03
		Inelastic heat fraction(β)	0.9
	Density(ρ)(kg/m^3^)	Workpiece(AISI 4340)	7850
		Tool(Carbide)	11500
	Elasticity	Workpiece	2.1 × 10^11^
		Tool	5.3 × 10^11^
	Conductivity(k) (W/m·k)	Workpiece	44.5
		Tool	120
	Specific heat(c) (J/kg·K)	Workpiece	502
		Tool	343.3
	Expansion(K^−1^)	Workpiece	1.23 × 10^−0.005^
		Tool	5.2 × 10^−0.006^
	Friction coefficient (μ)		0.2, 0.4, 0.6, 0.8
Process	Cutting speed (v) (m/s)		0.5, 1, 1.5, 2, 2.5
	Rake angle (γ) (°)		10°
	Clearance angle (α) (°)		10°

**Table 2:** Tool edge geometry in cutting tests with carbide tools.

Case	α_0_	α_1_	b_cf_
1	10	0	−
2	10	−10	0.0902
3	10	−15	0.1177
4	10	−20	0.1177
5	10	−25	0.1177
6	10	−30	0.1019
7	10	−35	0.0981

## Data Availability

This study did not report any data.

## List of Symbols

$\alpha_{1}$	Chamfer angle
$\alpha_{0}$	α_0_ Front angle
$b_{cf}$	lengths
σ	Equivalent stress
A	Initial yield stress
B	Hardening modulus
C	Strain rate dependency coefficient
ε	Plastic strain
$\varepsilon^{1}$	Strain rate
$\varepsilon_{0}{}^{1}$	Reference strain rate
T	Operating temperature
$T_{room}$	Room temperature
$T_{melt}$	Melting temperature
n	Strain hardening exponent
m	Thermal softening coefficient
μ	Coefficient of dynamic friction between tool and workpiece
